# Superoxide dismutase/catalase mimetic EUK-134 prevents diaphragm muscle weakness in monocrotalin-induced pulmonary hypertension

**DOI:** 10.1371/journal.pone.0169146

**Published:** 2017-02-02

**Authors:** Koichi Himori, Masami Abe, Daisuke Tatebayashi, Jaesik Lee, Håkan Westerblad, Johanna T. Lanner, Takashi Yamada

**Affiliations:** 1 Graduate School of Health Sciences, Sapporo Medical University, Sapporo, Japan; 2 Department of Physiology and Pharmacology, Karolinska Institutet, Stockholm, Sweden; Semmelweis Egyetem, HUNGARY

## Abstract

Patients with pulmonary hypertension (PH) suffer from inspiratory insufficiency, which has been associated with intrinsic contractile dysfunction in diaphragm muscle. Here, we examined the role of redox stress in PH-induced diaphragm weakness by using the novel antioxidant, EUK-134. Male Wistar rats were randomly divided into control (CNT), CNT + EUK-134 (CNT + EUK), monocrotaline-induced PH (PH), and PH + EUK groups. PH was induced by a single intraperitoneal injection of monocrotaline (60 mg/kg body weight). EUK-134 (3 mg/kg body weight/day), a cell permeable mimetic of superoxide dismutase (SOD) and catalase, was daily intraperitoneally administered starting one day after induction of PH. After four weeks, diaphragm muscles were excised for mechanical and biochemical analyses. There was a decrease in specific tetanic force in diaphragm bundles from the PH group, which was accompanied by increases in: protein expression of NADPH oxidase 2/gp91phox, SOD2, and catalase; 3-nitrotyrosine content and aggregation of actin; glutathione oxidation. Treatment with EUK-134 prevented the force decrease and the actin modifications in PH diaphragm bundles. These data show that redox stress plays a pivotal role in PH-induced diaphragm weakness. Thus, antioxidant treatment can be a promising strategy for PH patients with inspiratory failure.

## Introduction

Pulmonary hypertension (PH) is characterized by a progressive pulmonary vascular remodeling leading to high blood pressure in the pulmonary artery. Patients with PH suffer from dyspnea, which is associated with decreased exercise tolerance and hence results in the impairment of daily activity and quality of life. The dyspnea in PH patients has traditionally been attributed to primary abnormalities in pulmonary gas exchange [1]. However, clinical studies suggest a pivotal role of inspiratory muscle dysfunction in PH-induced dyspnea [2, 3]. This was further supported by recent work revealing a significant reduction in force generation in the diaphragm of patients [4] and an animal model [4–6] with PH.

The diaphragm force deficit in PH results from muscle wasting and intrinsic contractile dysfunction [4, 5]. Intriguingly, previous studies on skinned fibers consistently showed a reduction in isometric force normalized by cross sectional area (i.e. specific force) in PH patients and animals [4–6]. Thus, PH-induced diaphragm weakness is likely to result from myofibrillar dysfunction. Theoretically, myofibrillar force generation capacity is determined by the fraction of force generating cross-bridges and/or force per cross-bridge. In this regard, previous studies have demonstrated decreased maximal tension without reduction in myosin heavy chain content in diaphragm from PH rat [4, 5], suggesting a reduction in force per cross-bridge as a cause of PH-induced diaphragm dysfunction.

Patients with PH hyperventilate not only during exercise, but also under resting conditions [2]. Similarly, it was reported that breathing frequencies are markedly increased in rats with PH [4, 5]. Increased contractile activity has been shown to cause overproduction of superoxide (O^•^_2_^-^) through activation of mitochondrial respiration and NADPH oxidase 2 (NOX2) [7]. In the presence of the nitric oxide, O^•^_2_^-^ can be converted to peroxynitrite anion (ONOO^-^). Importantly, exposure of ONOO^-^ induces myofibrillar dysfunction in rat skinned fibers [8].

Manganese-salen complexes possess combined superoxide dismutase (SOD) and catalase mimetic function, and thus catalytically eliminate O^•^_2_^-^ and hydrogen peroxide (H_2_O_2_). A series of compounds (EUK) based on these SOD/catalase mimetics were shown to eliminate NO-derived radicals, including ONOO^-^ [9], and protect against redox stress in various neurological disorders, including Parkinson’s disease [10], Alzheimer’s disease [11], and stroke [12]. More interestingly, EUK-134 [manganese 3-methoxy *N*, *N*’-bis(salicylidene)ethylenediamine chloride] has recently been shown to prevent redox stress-induced muscle wasting and weakness in various pathological conditions [13–15].

Two rodent models, exposed to either hypoxia or monocrotaline (MCT), have been used to investigate the PH [16]. Although the hypoxia model has contributed to a better understanding of vascular remodeling in PH, it was suggested that this model does not develop the complex lesions found in patients with severe PH [17]. In contrast, the MCT model has been widely used to induce PH and to study skeletal muscle abnormalities caused by PH [4–6]. Thus, in the present study, we used the MCT-induced PH rats to test the hypothesis that PH-induced diaphragm force depression is mediated by redox stress. We demonstrated that the specific force depression in PH diaphragm was accompanied by hypernitration and aggregation of actin. These deleterious changes were prevented by the administration of the antioxidant EUK-134.

## Materials and Methods

### Ethical approval

All experimental procedures were approved by the Committee on Animal Experiments of Sapporo Medical University (No. 14–055). Animal care was in accordance with institutional guidelines.

### Experimental design

In order to investigate the role of oxidative stress in PH-induced diaphragm dysfunction, we performed two separate experiments.

#### Experiment 1

This experiment was performed to determine the effect of treatment with the antioxidant EUK-134 as such on the contractility of diaphragm in normal rats. Male Wistar rats (5 week old, n = 12) were supplied by Sankyo Labo Service (Sapporo, Japan). Rats were given food and water ad libitum and housed in an environmentally controlled room (24 ± 2°C) with a 12-h light-dark cycle. Rats were randomly assigned into control (CNT, n = 6) and CNT + EUK-134 (CNT + EUK, n = 6) groups. CNT and CNT + EUK animals received daily intraperitoneal administration of saline and EUK-134 (3 mg/kg body weight/day), respectively. This dosage and route of administration was similar to that used by Lawler et al. [14]. At the completion of the 4 weeks treatment periods, rats were killed by cervical dislocation under isoflurane anesthesia and a diaphragm fiber bundle was dissected from each animal. Our results demonstrate that treatment with EUK-134 did not affect diaphragm contractility in normal rats (see Results). Thus, we next performed a second experiment to define the role of oxidative stress in diaphragm dysfunction in PH rats.

#### Experiment 2

To assess whether oxidative stress is involved in the mechanism underlying PH-induced diaphragm dysfunction, rats (5 week old, n = 25) were divided into control (CNT, n = 7), MCT-induced PH (n = 8), and PH + EUK-134 (PH-EUK, n = 10) groups. PH was induced by a single intraperitoneal injection of MCT (60 mg/kg body weight). EUK-134 (3 mg/kg body weight/day) was administered daily intraperitoneally for four weeks beginning the day after MCT injection. Following the experimental period, rats were sacrificed and the diaphragm muscle was excised.

### Measurement of isometric contractile force

The diaphragm fiber bundle was mounted between a force transducer (Nihon Kohden) and an adjustable holder. The bundle was superfused with Tyrode solution (mM): NaCl, 121; KCl, 5; CaCl_2_, 1.8; MgCl_2_, 0.5; NaH_2_PO_4_, 0.4, NaHCO_3_, 24; EDTA, 0.1; glucose, 5.5. The solution was bubbled with 5% CO_2_-95% O_2_, which gives an extracellular pH of 7.4, and kept at 30°C. Supramaximal, 0.5 ms current pulses were applied via two platinum plate electrodes placed on each side of the muscle. Muscle length was adjusted to the length (*L*_0_) giving maximum tetanic force. The force-frequency relationship was determined by evoking tetani at different frequencies (10–120 Hz, 600 ms duration) at 1 min intervals. Absolute force was normalized to cross-sectional area, calculated as muscle weight divided by *L*_o_ and density (1056 kg m^-3^).

### Immunoblots

Immunoblots were performed using: anti-NADPH oxidase 2 catalytic subunit gp91^phox^ (NOX2/gp91^phox^) (Abcam); anti-neuronal, -endothelial, and -inducible nitric oxide synthases (nNOS, eNOS, and iNOS, respectively) (610308, 610296, 610328, respectively, BD Biosciences); anti-manganese SOD (SOD2) (06–984, Upstate); anti-catalase (C0979, Sigma); anti-tumor necrosis factor α (TNF-α) (11948, Cell Signaling); anti-high mobility group box 1 (HMGB1) (326059652, SHINO-TEST); anti-ryanodine receptor 1 (RyR1) (MA3-925, Thermo); anti-3-nitrotyrosine (3-NT) (ab52309, Abcam); anti-troponin T (TnT) (T6277, Sigma); anti-actin (A4700, Sigma); anti-dihydropyridine receptor α2 subunit (DHPR) (ab2864, Abcam); anti-sarcoplasmic reticulum (SR) Ca^2+^-ATPase (SERCA) 1 (MA3-911, Thermo); anti-SERCA2 (MA3-919, Thermo).

Muscle pieces were homogenized in ice-cold homogenizing buffer (40 μl/mg wet wt) consisting of (mM): Tris maleate, 10; NaF, 35; NaVO_4_, 1; 1% Triton X 100 (vol/vol), and 1 tablet of protease inhibitor cocktail (Roche) per 50 ml. To extract myofibrillar proteins, an aliquot of homogenized muscle was centrifuged at 4°C for 15 min at 14,000 g. The supernatant was discarded and the resulting myofibrillar enriched pellet was resuspended in ice-cold high-salt buffer (40 μl/mg wet wt) consisting of (mM): 300 NaCl, 100 NaH_2_PO_4_, 50 Na_2_HPO_4_, 10 Na_4_P_2_O_7_, 1 MgCl_2_, 10 EDTA, pH 6.5 and 1 tablet of protease inhibitor cocktail (Roche) per 50 ml. The protein content was determined using the Bradford assay [18].

Aliquots of the muscle homogenates (20 μg) were then diluted with SDS-sample buffer (mM): Tris HCl, 62.5; 2% SDS (wt/vol); 10% glycerol (vol/vol); 5% 2-mercaptoethanol (vol/vol); 0.02% bromophenol blue (wt/vol). For the detection of 3-NT and actin, muscle homogenates (20 μg) were diluted with non-reducing Laemmli buffer (mM): urea, 4000; Tris, 250; 4% SDS (vol/vol); 20% glycerol (vol/vol); 0.02% bromophenol blue (wt/vol). Proteins were applied to a 4–15% Criterion Stain Free gel (BioRad, Hercules, CA). Gels were imaged (BioRad Stain Free imager), and then proteins were transferred onto polyvinylidine fluoride membranes. Membranes were blocked in 3% (wt/vol) non-fat milk, Tris-buffered saline containing 0.05% (vol/vol) Tween 20, followed by incubation with primary antibody, made up in 5% (wt/vol) non-fat milk overnight at 4°C. Membranes were then washed and incubated for 1 hours at room temperature (~23°C) with secondary antibody (1:5000, donkey-anti-rabbit or donkey-anti-mouse, Bio-Rad). Images of membrane were collected following exposure to chemiluminescence substrate (Millipore) using a charge-coupled device camera attached to ChemiDOC MP (Bio-Rad), and Image Lab Software (Bio-Rad) was used for detection as well as densitometry.

### MyHC isoforms separation

Aliquots of the extracts containing 5 μg myofibrillar protein were applied for MyHC electrophoresis as previously described in detail [19]. Using a 6% polyacrylamide slab gel, electrophoresis was run at 4°C for 48 hours at 160 V and stained with Coomassie brilliant blue. Images of gels were densitometrically evaluated with ImageJ.

### Glutathione contents

The amounts of total, reduced (GSH), and oxidized glutathione (GSSG) were determined by spectrophotometry methods, as described by Baker et al. [20]. Diaphragm muscles, weighing ~30 mg, were minced, placed on ice in nine volumes of 5% (wt/vol) 5-sulfosalicylic acid for 30 mins, and then centrifuged at 16,000 *g* for 10 mins to remove precipitated materials. For total glutathione (GSH + GSSG), triethanolamine was added to the supernatant to give a final concentration of 6% (vol/vol). For GSSG measurements, 2% (vol/vol; final concentration) 2 vinylpyridine was additionally added. The assay buffers contained 1.52 mM NaH_2_PO_4_, 7.6 mM Na_2_HPO_4_, 0.485 mM EDTA, 1 U/ml glutathione reductase, and 0.1 mM NADPH (pH 7.5). After the addition of an aliquot of the sample, the assay mixture was incubated for 2 mins. The reaction was started by adding 5,5’-dithiobis-(2-nitrobenzoic acid) to give a final concentration of 0.4 mM. The glutathione concentration was determined spectrophotometrically at a wavelength of 412 nm. The GSH content was calculated as the difference between total glutathione and GSSG contents.

### Statistics

Data are presented as mean ± SD. Student’s unpaired *t* test was used to detect differences between PH and CNT muscles. A two-way variance analysis was performed to evaluate the influence of PH and EUK-134. The Bonferroni *post hoc* test was used to isolate the significantly different means. A *P* value less than 0.05 was regarded as statistically significant.

## Results

### EUK-134 does not alter diaphragm contractile function in normal rats

There was no difference in body and heart weights between CNT and CNT + EUK group (249 ± 16 versus 260 ± 9 g [n = 6]; *P* > 0.05; 664 ± 45 versus 675 ± 49 mg [n = 6]; *P* > 0.05). Moreover, compared to CNT animals, the specific force (i.e. force per cross-sectional area) was not changed in CNT + EUK animals at any stimulation frequencies from 1 Hz to 120 Hz (*see* S1 Fig). Thus, treatment with EUK-134 as such did not affect the contractile function of diaphragm muscles. No further experiments were performed on these groups.

### Monocrotaline induces cardiac hypertrophy

The body weights of the PH group were significantly lower than those of the CNT group (Table 1). Similar to what was shown in a previous study [21], MCT treatment resulted in a significant increase in the right ventricle (RV) weights (both absolute and normalized to heart weights), demonstrating the successful induction of PH. EUK-134 treatment did not prevent the change in body or RV weights of PH rats.

**Table 1:** Body and heart weights.

	CNT (n = 7)	PH (n = 8)	PH+EUK (n = 10)
Bwt (g)	248 ± 19	171 ± 38^*^	176 ± 29^*^
Hwt (mg)	664 ± 63	691 ± 112	680 ± 105
Hwt/Bwt (mg/g)	2.68 ± 0.1	4.11 ± 0.49^*^	3.90 ± 0.41^*^
RVwt	117 ± 7	206 ± 49^*^	202 ± 65^*^
RVwt/Bwt (mg/g)	0.47 ± 0.21	1.22 ± 0.21^*^	1.15 ± 0.31^*^
RVwt/Hwt (mg/mg)	0.18 ± 0.01	0.30 ± 0.04^*^	0.29 ± 0.06^*^

Values are means ± SD for 7–10 rats in each group. CNT, control; PH, monocrotaline-induced pulmonary hypertension; EUK, treatment with EUK-134; Bwt, body weight; Hwt, Heart weight; RVwt, right ventricle weight.

*P < 0.01, compared with CNT.

### Specific force depression is accompanied by upregulation of redox enzymes in diaphragm from PH rat

**Fig 1:**
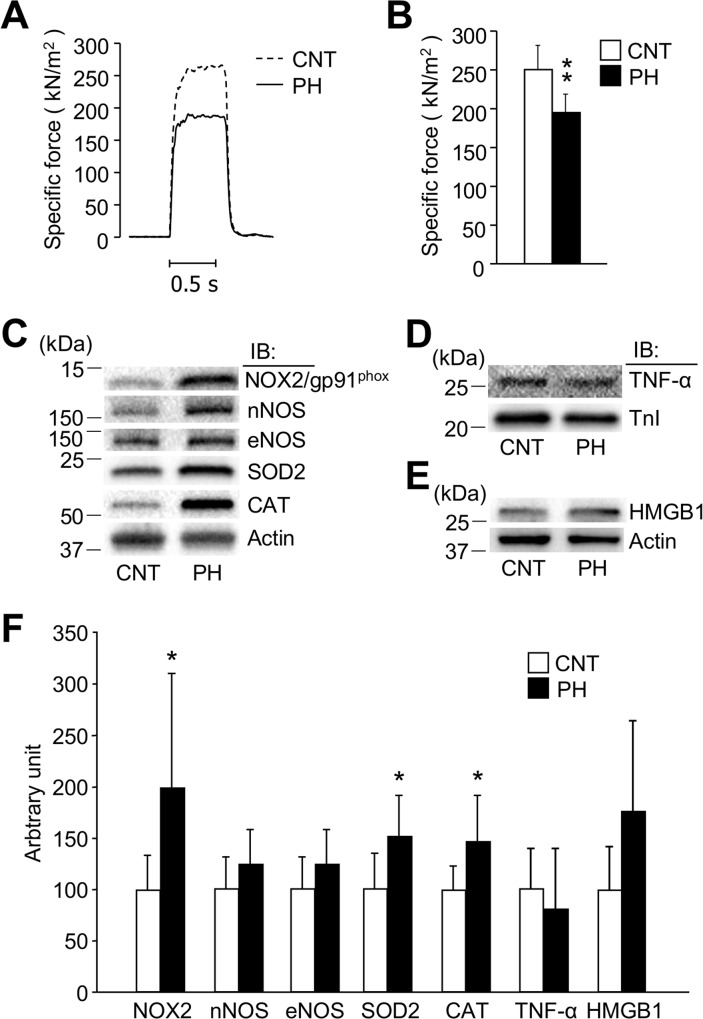
Specific force depression is accompanied by upregulation of redox enzymes in diaphragm from PH rat. *A*: typical examples (100 Hz stimulation frequency, 600 ms train duration) of specific force in diaphragm fiber bundles from CNT and PH rats. *B*: Specific force at 100 Hz stimulation frequency. *C*-*E*: representative western blots illustrating the levels of NADPH oxidase (NOX2/gp91^phox^), neuronal nitric oxide synthase (nNOS), endothelial NOS (eNOS), superoxide dismutase 2 (SOD2), catalase (CAT), tumor necrosis factor (TNF)-α and high mobility group box 1 (HMGB1). Inducible NOS (iNOS) was not detected in either group. *F*: quantification of the levels of redox-related proteins and inflammatory mediators normalized to actin or troponin I (TnI) content. Data show mean ± SD for 5–8 rats in each group. ^*^P < 0.05, ^**^P < 0.01 *vs*. CNT.

In agreement with previous studies, MCT administration induced contractile dysfunction in the diaphragm. Maximum specific tetanic force (100 Hz stimulation frequency) was ~20% lower in PH muscles than in CNT muscles (Fig 1A and 1B).

To further investigate the mechanism underlying the PH-induced muscle dysfunction, we compared the expression of redox enzymes and inflammatory mediators in PH and CNT diaphragm muscles. The expressions of NOX2/gp91^phox^, but not nNOS and eNOS, were significantly increased by ~100% in PH muscles (Fig 1C and 1F). iNOS was not detected in either group. Moreover, the protein expression of SOD2 and catalase were significantly increased in muscles from the PH rats.

The inflammatory mediators TNF-α and HMGB1 can induce redox stress [22, 23]. The protein expression of TNF-α and HMGB1 was not significantly different between PH and CNT muscles (Fig 1D–1F).

### Neither the expression of contractile proteins nor proteins involved in excitation-contraction coupling are altered in diaphragm from PH rats

**Fig 2:**
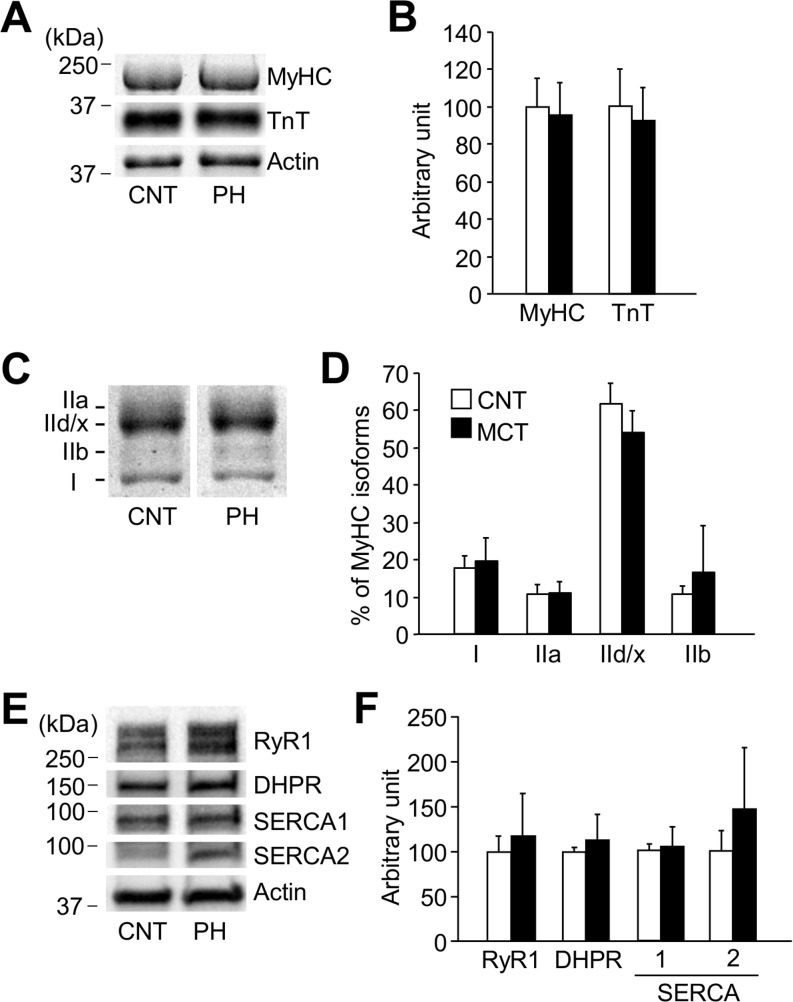
Neither contractile proteins nor excitation-contraction coupling proteins are altered in PH diaphragm muscles. *A*: Stain free images of myosin heavy chain (MyHC) and western blots of troponin (Tn) T. *B*: the expression levels of MyHC or TnT normalized to actin content. *C*: electrophoretically separated MyHC isoforms. *D*: percentage distribution of MyHC isoforms: I, slow myosin isoform; IIa, IId/x, and IIb, fast myosin isoforms. *E*: representative western blots of ryanodine receptor (RyR1), dihydropyridine receptor α2 subunit (DHPR), sarcoplasmic reticulum Ca^2+^-ATPase (SERCA) 1, and SERCA2. *F*: the expression levels of RyR1, DHPR, SERCA1, or SERCA2 normalized to actin content. Control (CNT), white bars; AIA, black bars. Data represent mean ± SD for 4–8 rats in each group.

There was no difference in total myofibrillar protein concentration in diaphragm muscles between CNT and PH rats (92.9 ± 15.3 versus 85.9 ± 18.5 mg/g wet weight [n = 7–8]; *P* > 0.05). A selective loss of myofibrillar proteins, such as myosin [24] and TnT [25], has been implicated in the muscle weakness in pathological conditions. However, neither the MyHC nor TnT content differed between PH and CNT muscles (Fig 2A and 2B). Moreover, the distribution of MyHC isoforms was not altered in PH muscles (Fig 2C and 2D). There were no differences in the expression levels of the Ca^2+^ handling proteins RyR1, DHPR, SERCA1, or SERCA2 between the groups (Fig 2E and 2F).

### EUK-134 protects against PH-induced diaphragm force depression

**Fig 3:**
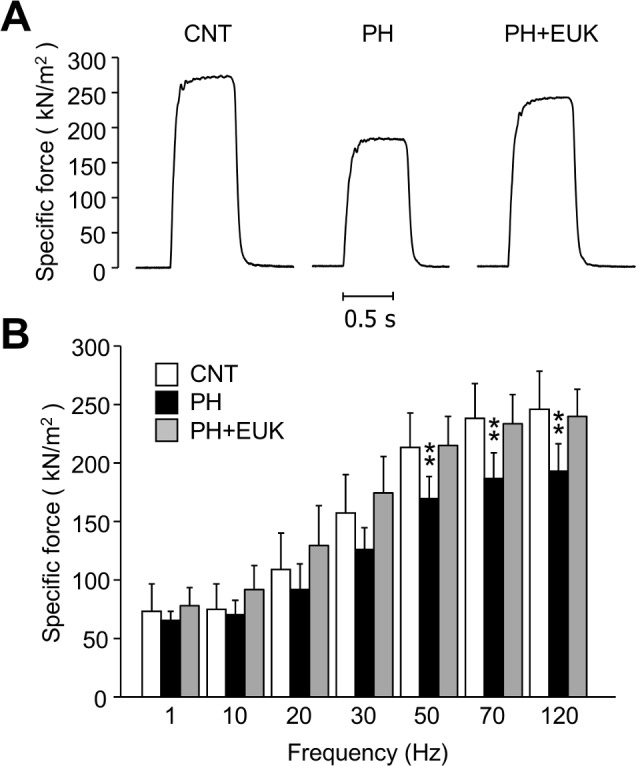
Antioxidant treatment prevents contractile dysfunction in PH diaphragm muscles. *A*: typical examples (120 Hz stimulation frequency, 600 ms train duration) of specific force in diaphragm fiber bundles from CNT and PH rats with or without EUK-134 (EUK) treatment. *B*: specific force-frequency relationship. Data show mean ± SD for 7–10 rats in each group. ^**^P < 0.01 *vs*. CNT.

It is generally accepted that redox stress depresses the intrinsic force production in rat diaphragm fibers [26]. Therefore, we tested whether treatment with an antioxidant could prevent the PH-induced diaphragm weakness. The results show that daily administration of EUK-134 prevented the PH-induced decrease in specific force in diaphragm (Fig 3), despite having no obvious effect on the development of cardiac hypertrophy (Table 1).

### EUK-134 prevents the oxidation of glutathione in diaphragm from PH rat

**Fig 4:**
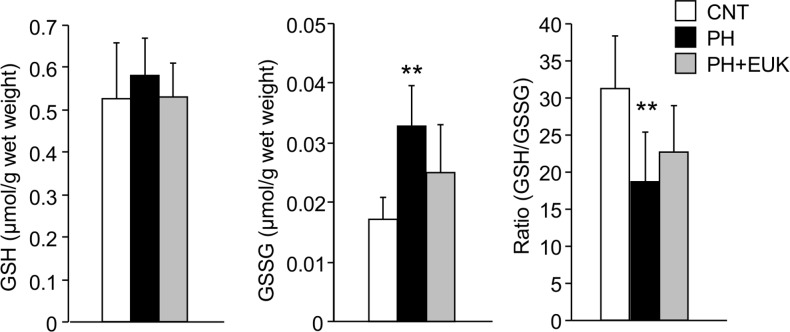
GSH: GSSG ratio is decreased in diaphragm muscles from PH rat. The cytoplasm levels of GSH, GSSG, and the GSH: GSSG ratio in diaphragm muscles from CNT and PH rats with or without EUK-134 (EUK) treatment. Data show mean ± SD for 5–8 rats in each group. ^**^P < 0.01 *vs*. CNT.

The cytoplasm levels of GSH and GSSG were determined in diaphragm muscle from CNT, PH, and PH+EUK group (Fig 4). There was no difference in the amount of GSH between the groups. In contrast, the amount of GSSG was ~twofold higher in PH than in CNT muscles. Thus, the ratio of GSH: GSSG was markedly lower in the PH muscles. The amount of GSSG and the ratio of GSH: GSSG were not altered in PH+EUK muscles compared to CNT muscles.

### EUK-134 inhibits hypernitration and aggregation of actin in diaphragm muscles from PH rat

**Fig 5:**
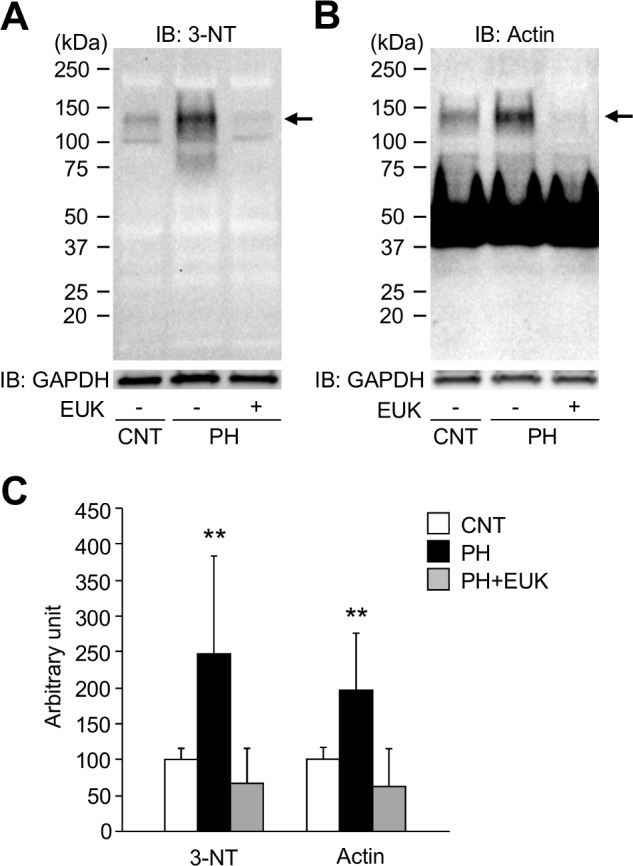
3-nitrotyrosine content is increased in actin aggregates from PH diaphragm muscles. *A* and *B*: representative western blots for 3-nitrotyrosine (3-NT) and actin in diaphragm muscles from CNT and PH rats with or without treatment with EUK-134 (EUK). *C*: intensities for the protein band at ~130 kDa (indicated by *arrows*) in 3-NT and actin were normalized to the glyceraldehyde-3-phosphate dehydrogenase (GAPDH) content. Data show mean ± SD for 6 rats in each group. ^**^P < 0.01 *vs*. CNT.

Previous studies have implicated redox modifications on myofibrillar protein as one of the mechanisms underlying the intrinsic contractile dysfunction of skeletal muscle in pathological conditions [15, 24, 27]. Thus, we next investigated whether the contractile dysfunctions were accompanied by myofibrillar redox modifications in the PH diaphragm muscle. Western blotting showed a specific increase in 3-NT at ~130 kD in PH diaphragm muscles (Fig 5A and 5C). Moreover, in addition to the normal actin band seen at ~40 kDa, there was a marked increase in an actin positive band at ~130 kDa in PH muscles (Fig 5B and 5C). Importantly, EUK-134 treatment prevented the hypernitration and aggregation of actin in PH diaphragm muscles.

## Discussion

In the present study, we show that PH induces depression in specific forces of rat diaphragm muscles. This contractile dysfunction was accompanied by the hypernitration and aggregation of actin. These changes were prevented by the daily administration of an antioxidant, EUK-134. Thus, our findings suggest a critical role for redox stress in PH-induced diaphragm dysfunction.

Decreased force production in skeletal muscle can arise from 1) reduced Ca^2+^ release from the SR, 2) decreased myofibrillar Ca^2+^ sensitivity, and/or 3) impaired ability of cross-bridges to generate force [28]. In the present study, depressed specific force production was found especially in high-stimulation frequencies in PH diaphragm (i.e. 50–120 Hz). Both reduced SR Ca^2+^ release and decreased myofibrillar Ca^2+^ sensitivity have little impact on force at high frequencies because of the non-linear relationship between force and [Ca^2+^] [29], which is flat at high but steep at low stimulation frequencies. Impaired cross-bridge force generation is therefore a likely mechanism underlying PH-induced diaphragm weakness. In support of this, a depression in specific force production was found in skinned diaphragm fibers from patients [4] and animal model [4–6] with PH.

A key finding of the present study is that the contractile dysfunction is accompanied by actin aggregates in PH diaphragm. We previously showed that actin aggregates is formed by disulfide bonds in skeletal muscle from adjuvant-induced arthritis rat [15]. The concomitant increase in 3-NT in aggregated actin indicates an excessive production of ONOO^-^ in diaphragm fiber from PH rat, because 3-NT is a product of tyrosine nitration mediated by ONOO^-^. Furthermore, ONOO^-^ can reversibly oxidize thiols to disulfide bonds [30], hence aggregation of actin is most likely induced by ONOO^-^ in PH diaphragm. In support of this, previous study showed that the globular (G)-actin polymerization and fibrillary (F)-actin depolymerization is sensitive to ONOO^-^ and an imbalance in the F-actin/G-actin equilibrium accounts for the impairment of myofibrillar function under ONOO^—^induced redox stress in pathophysiological conditions [31]. Thus, it appears that myofibrillar dysfunction is caused by redox modifications of actin in diaphragm muscle from PH rat.

Intriguingly, administration of the antioxidant EUK-134 completely protects against actin aggregates and contractile dysfunction in PH diaphragm. EUK-134 has catalytic activities of SOD and catalase [32], which enable this compound to metabolize O^•^_2_^-^ and convert the resultant H_2_O_2_ to water. In addition, this compound catalyzes the removal of ONOO^-^ [9]. Thus, our data indicate that EUK-134 reduced the production of ONOO^—^derived radicals, which counteracted to formation of actin aggregates and hence prevented the PH-induced contractile deficit in diaphragm muscles.

The selective loss of myofibrillar proteins, including myosin [33] and troponin T [25], was suggested to be involved in myofibrillar dysfunction in pathological condition. However, previous studies have shown that the expression levels in myosin[5, 6] and troponin T [6] are not decreased in diaphragm from PH animals. Our present data also showed no change in the expression levels of these myofibrillar proteins, demonstrating that the reduction in specific forces observed in PH diaphragm are not due to loss of myofibrillar proteins.

In agreement with the study by Manders et al. [5], we observed an increased breathing frequencies in PH rats (data not shown). Excessive exercise has been shown to enhance the production of O^•^_2_^-^ and NO, leading to the formation of ONOO^-^ in skeletal muscle [34]. Recent studies indicate that NOX2 is one of the critical source of O^•^_2_^-^ in this process [7, 35]. Our observation of increased NOX2/gp91phox, a membrane bound essential subunit of NOX2, expression therefore implies this enzyme as a source of O^•^_2_^-^ in PH diaphragm. Skeletal muscle constitutively expressed both nNOS and eNOS. Among them, increased rate of NO production during contraction was shown to be mediated by nNOS in mouse skeletal muscle [36]. Although we could not see any alterations in the expression levels of nNOS in PH diaphragm, chronic increase in contractile activity may increase NO production by an activation of this enzyme. In contrast, inflammatory mediators, such as TNF-α [37] and HMGB1 [22], have been suggested to induce redox stress in diaphragm muscle. The expression levels of these proteins were, however, not changed in PH diaphragm. Thus, the hyperpnea-induced increased contractile activity, rather than inflammatory signaling, is likely to cause increased ONOO^-^ production in PH diaphragm muscles.

Redout et al. [38] reported that EUK-134 treatment improves systolic function in monocrotaline-induced PH rats. Due to practical reasons, we could not measure cardiac function in PH animals in the present study, but the present finding that EUK-134 treatment did not prevent the marked increase in normalized RV weight in PH rats speaks against a protective effect on cardiac function. The reason for this discrepancy is not clear but could be explained by the difference in the levels of redox stress between cardiac and diaphragm muscles in PH rats. Indeed, Redout et al. [38] used about 8-fold higher dose for EUK-134 than the present study (i.e. 25 mg/kg vs 3 mg/kg). Thus, it appears that our dosage of EUK-134 is not high enough to prevent PH-induced RV remodeling.

## Study Limitations

In this initial study, EUK-134 was administered daily starting one day after induction of PH and it then effectively prevented the development of diaphragm weakness. However, it is unclear as to whether EUK-134 can reverse diaphragm dysfunction, i.e. if it is effective when administered after the induction of weakness. Thus, considering the clinical values of the present work, subsequent studies should be performed where EUK is administered after the onset of PH.

## Conclusions

The present study demonstrates using MCT-induced PH rats that PH causes diaphragm weakness, presumably by inducing myofibrillar dysfunction resulting from redox modifications of actin. These changes were prevented by daily administration of the antioxidant EUK-134. Thus, the EUK series of compounds may become valuable therapeutic agents to counteract muscle weakness in pathological conditions with oxidative stress, including PH patients with inspiratory failure.

## Supporting Information

S1 Fig EUK-134 does not alter diaphragm contractile function in normal rats. Specific force-frequency relationship in diaphragm fiber bundles from control (CNT) rats with or without EUK-134 (EUK) treatment. Data show mean ± SD for 6 muscles in each group.
(TIF)
