# Supplementary figures and images for: Superoxide dismutase/catalase mimetic EUK-134 prevents diaphragm muscle weakness in monocrotalin-induced pulmonary hypertension
Source: PLoS One. 2017 Feb 2;12(2):e0169146. doi: 10.1371/journal.pone.0169146 (PMC5289453; doi:10.1371/journal.pone.0169146)

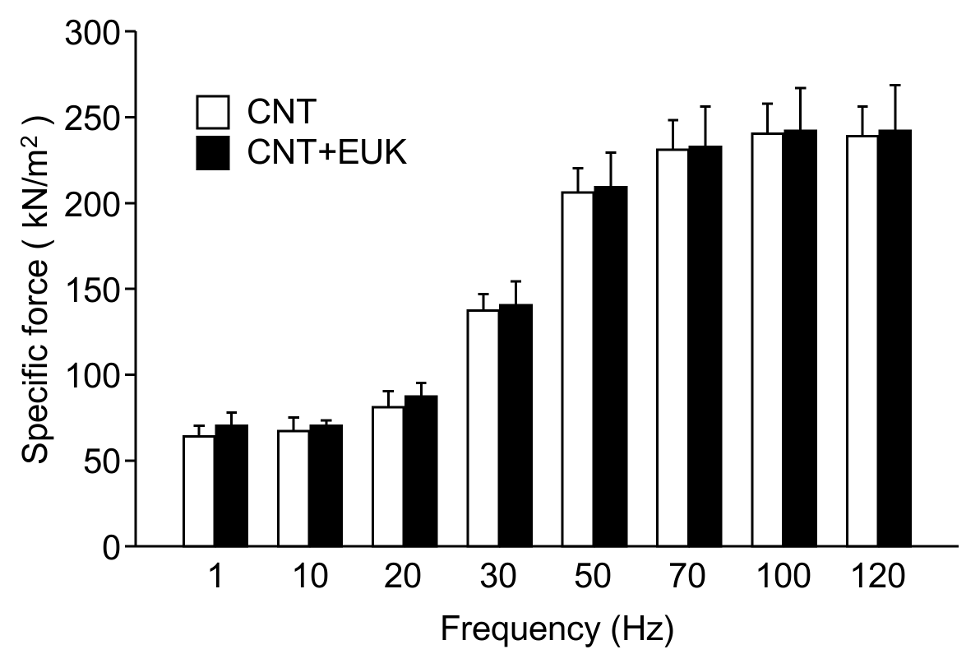

Supplement: S1 Fig — Specific force-frequency relationship in diaphragm fiber bundles from control (CNT) rats with or without EUK-134 (EUK) treatment. Data show mean ± SD for 6 muscles in each group. (TIF) [file pone.0169146.s001.tif]
